# Air Flow Detection in Crude Oil by Infrared Light

**DOI:** 10.3390/s17061278

**Published:** 2017-06-03

**Authors:** Guilherme Dutra, Cicero Martelli, Marco José Da Silva, Rodolfo L. Patyk, Rigoberto E. M. Morales

**Affiliations:** 1 Graduate Program in Electrical and Computer Engineering (CPGEI), Federal University of Technology-PR, Curitiba 80230-901, Brazil; gdutra@alunos.utfpr.edu.br (G.D.); mdasilva@utfpr.edu.br (M.J.D.S.); rodolfopatyk@gmail.com (R.L.P.); 2 Department of Electronics, Federal University of Technology-PR, Curitiba 80230-901, Brazil; 3 Graduate Program in Mechanical and Materials Engineering (PPGEM), Federal University of Technology-PR, Curitiba 80230-901, Brazil; rmorales@utfpr.edu.br; 4 Multiphase Flow Research Center (NUEM), Federal University of Technology-PR, Curitiba 80230-901, Brazil

**Keywords:** infrared radiation, optical detection, two-phase flow, crude oil-air flow, absorption

## Abstract

In this paper, we used infrared light in the range of 8–12 μm to develop and test an optical imaging system to detect air bubbles flowing in oil. The system basically comprises a broadband light source and a 31 × 32 thermopile array to generate images. To analyze the effects related to light absorption, reflection, and refraction on air-oil boundaries, a numerical model was developed and the predominance of the refraction instead of the absorption in bubbles with diameters below a certain critical value was observed. The IR region of the electromagnetic spectrum has both optical and thermic behavior. To understand the limits of each effect on the oil flow imaging, a study of the influence of temperature variation on the petroleum optical detection was performed. The developed optical imaging system allowed the detection of air flow in static oil and in oil-air two-phase flow. With the presented system, it was possible to achieve images through up to 12 mm of oil volumes, but this may be enhanced by the use of optimized IR sources and detectors.

## 1. Introduction

Some of the most important industrial activities in the world are the production, transport and refining of crude oil. Not only are the majority of transport systems around the world based on petroleum derivatives, but also the production of synthetic materials uses various petroleum derivatives which are used in many different areas of our lives. Given the growing necessity to increase efficiency in this industry, there is a constant need to develop sensors with the capability of measuring more accurately and in more challenging locations a greater number of variables throughout the oil production chain. An example of the complexity of the production and extraction of crude oil is presented in the pre-salt region exploration in Brazil, where the oil reservoirs are at about 5–6 km in depth and located 300–350 km off the coast [1].

Crude oil has a very complex chemical composition since it is composed by hundreds of different molecules, particularly hydrocarbons, and each of them has specific physicochemical properties [2]. From the optical viewpoint, considering the visible spectrum, petroleum is opaque and it is not possible to “see” through it unless it is deposited as a thin film on a glass substrate, for example. It turns out, however, that virtually all oils show some level of transparency in the infrared region of the electromagnetic spectrum. This is well known to chemists who have been using this region of the electromagnetic spectrum to characterize and study samples of crude oils from wells around the world for decades [3]. This type of measurement and characterization are performed in a controlled laboratory environment.

The use of an optical technique as a measurement system or a maintenance tool has advantages when compared to other methods. First, it is nonintrusive, so does not disturb the flow in wells and pipelines or obstruct them to the traffic of in-line maintenance tools. Optical detection systems can be made small, mechanically and thermally robust and, in general, have low power consumption. By scanning wavelengths, it is possible to add functionalities by implementing spectroscopy based chemical detection that can directly lead to chemical imaging and/or chemical species tomography [4,5], among other opportunities. Here we focus on the development of a technique to detect and study flows in crude oil, especially those with more than one phase.

There is a considerable number of different measurement techniques used and developed to investigate multiphase flows such as: conductive and capacitive probes [6,7], X-ray [8,9], ultrasound transducers [10,11], wire-mesh sensors [12,13], and guided [14,15] and unguided [16,17] optical methods.

Thus, the aim of the work presented here is to develop optical instrumentation capable of imaging inside crude oil volumes, either static or flowing in pipes, with the potential to be applied in the field. The optical transparency of crude oil at the infrared region of the electromagnetic spectrum creates an opportunity to develop high spatial resolution spectroscopic techniques that allow for the development of advanced tools for studying multiphase flow phenomena.

## 2. Materials and Methods

Petroleum is opaque in the visible region of the electromagnetic spectrum and therefore it is common sense that optical techniques cannot be used as a noninvasive tool to monitor any physical or chemical phenomena through a petroleum volume. There is, however, an opportunity when the infrared region of the petroleum optical absorption spectrum is considered [18] given that it is semitransparent and therefore optical radiation can propagate through a volume containing petroleum or a mixture with petroleum.

Table 1 shows the transmittances of the crude oil sample used in the experiments. The oil sample was provided by Petrobras and comes from the Brazilian shores. The measurements were carried out using FTIR (Fourier transform infrared spectroscopy) and results show five distinct regions of the spectrum. It is possible to observe the large difference between the transmission coefficients for the visible region, 0.5 μm (Region 1), in comparison to that for the region between 8 and 12 μm (Region 5).

**Figure 1:**
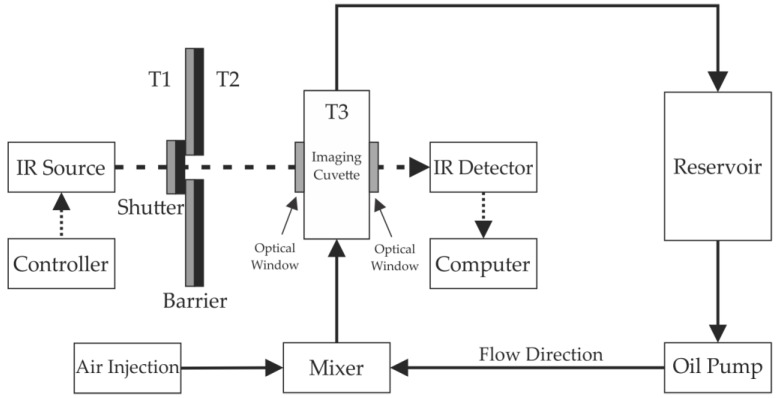
Experimental setup with IR detection and crude oil-air flow.

The experimental setup developed for flow measurements as well as for the optical and thermal characterization is shown in Figure 1. For the infrared source, a 1000 W Quartz Tungsten Halogen Lamp (Newport 6317) (Oriel®, Santa Clara, CA, USA) was used, centered at 1.2 μm, housed on a Newport 66885 Research QTH Lamp Housing (Oriel®, Santa Clara, CA, USA), and controlled by the Newport 69935 QTH Lamp Power Supply. A HEIMANN HTPA 31 × 32 thermopile array operating in the region between 8 and 12 μm was utilized as the infrared detector. The detector was housed in a metal module and has an acquisition rate of 10 frames per second and a resolution of 992 pixels. A cuvette was developed using two optical windows to allow the IR radiation to interact with the fluid whilst ensuring a continuous flow. The optical windows are made of borosilicate microscope coverslips. The length of the interaction between the fluid and the IR radiation is 12 mm. The thermocouples T1, T2, and T3 measure the temperature of the IR source, the environment and the crude oil inside of the cuvette, respectively.

The two-phase flow occurs in a closed loop where the oil is pumped mechanically from a reservoir to the imaging cuvette. The air is injected near the cuvette and, after interacting with the IR radiation, separated from the oil by gravity in the reservoir. The interaction region is positioned to allow the vertical flow from bottom to top.

Besides generating infrared radiation, the halogen lamp produces heat, increasing the temperature in all surroundings. As the temperature increases, the IR radiation emitted by all components and parts increases, enhancing the background noise at the IR detector. This is mitigated by using appropriated barriers that limit the IR radiation uptake to the cuvette. The barrier has low emissivity, high reflectivity, and low absorption materials on the source side in order to reflect the radiation, and high emissivity, low reflectivity, and high absorption material on the detector side in order to absorb, but not to reflect and not to emit undesirable radiations. The shutter allows the flux of radiation only when it is desired.

## 3. Results and Discussion

### 3.1. Temperature Monitoring

As mentioned above, the increasing temperature of the system can interfere with the measurement. To understand the behavior of the system and its response to temperature variations, three thermocouples, type k, were positioned (Figure 1): (T1) to measure the temperature stabilization of the source, (T2) to measure the ambient temperature near the interaction region, and (T3) to measure the crude oil temperature. 

**Figure 2:**
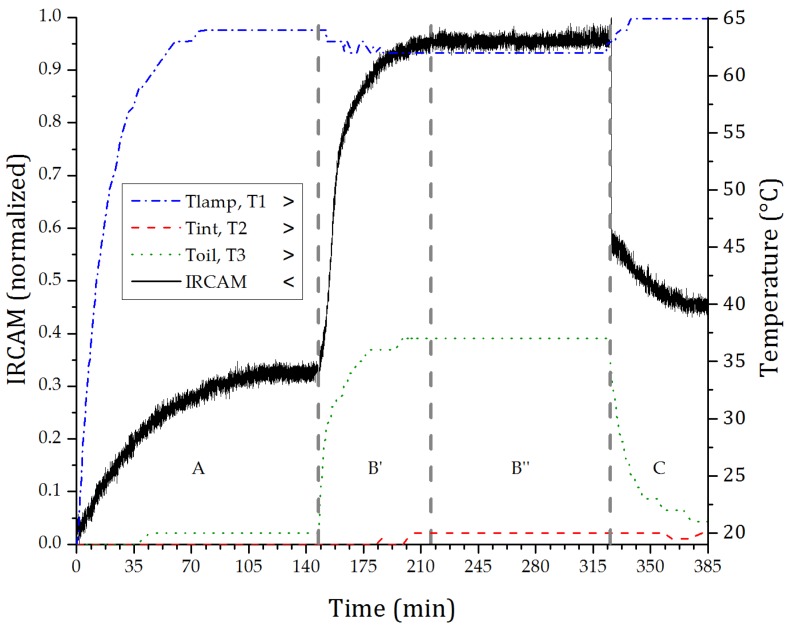
Optical response of the IR detector in relation to the heating of the crude oil sample due to the incidence of the IR radiation source.

This measurement is performed with static crude oil inside of the imaging cuvette, and the IR source is started with the shutter closed, as shown in Figure 2. At 150 min, the lamp and the IR detector reach the thermal equilibrium, then the shutter is opened, allowing the light to irradiate the imaging cuvette. At 330 min, the shutter is closed and the temperature data is acquired for another 60 min.

The plot in Figure 2 analyzes the thermic and optical behavior of the system, which is divided into four regions: (A) the thermal stabilization of the system after the source is switched on while the shutter remains closed; (B′) the shutter is open; (B″) the thermal stabilization of the system with the open shutter; and (C) the shutter is closed.

The response of the IR detector is represented by the solid line in Figure 2. In region (A), the effect of heating the environment and its impact in the detected signal can be observed. At (B), it is perceived that the first component refers to the light transmission through the sample reaches the detector directly with an abrupt increase in intensity at the opening of the shutter, followed by a slow component referring to the heating of the sample because of the IR radiation absorption by the sample. It is important to point out that the optical source has a broad spectrum covering from the visible (VIS) to mid-infrared (MIR). In (B″), the thermal stability of the sample is observed as well as the irradiation level that reaches the detector. In (C), the shutter is closed and it is possible to see the sharp fall of the signal due to the blockage of the radiation, followed by the cooling of the sample. The variation that appears between the end of region (A) and the end of the plot accounts for the increase in temperature of the whole system during the measurement.

The dashed-dot trace of Figure 2 shows the temperature at the output of the IR source. In (B′) and (B″) there is a drop in temperature due to the opening of the shutter. Likewise, the shutter is covered by a material with high reflectivity. Thus, part of the signal emitted by the source is reflected, thereby increasing the temperature in the lamp output in regions (A) and (C). In Figure 2, the dashed trace represents the room temperature, which is slightly altered during the test. As expected, the environment temperature does not have any abrupt change with the opening or closing of the shutter. The temperature of the sample, shown by the dotted line, suffers a slight increase before the shutter is opened. After the opening, it undergoes a rapid increase up to the stabilization and it then decays with the shutter closing.

### 3.2. Bubble Diameter Effect on Optical Detection

Basically, the optical detection principle is governed by Beer-Lambert’s law, but there are other effects that occur with the radiation and need to be taken into consideration. Electromagnetic radiation, when propagating through a material medium, can withstand absorption and scattering. If this radiation propagates from one medium to another it can also undergo refraction. The Snell’s law determines the angle of reflection and refraction, and the Fresnel’s coefficients determine what percentage of the radiation will be refracted and which will be reflected [19]. Hence, considering the circular shape of the air bubbles in oil, it is expected that a bubble flowing through the optical path of the radiation can function as a convergent or divergent lens, depending on its refractive index and diameter. To verify this lensing effect, a 2D ray trace numerical model taking into account reflection, refraction, and absorption of radiation was developed.

In the model, the radiation emitted by a point source is considered to have a numerical aperture = 9°, originating from point (0, 0) and composed by *n* rays. Each ray has unitary intensity and is separately analyzed and only those rays that arise at the detector are integrated. The detector has a finite length *d_PhD_*, is plane and is centered in the position (*x_PhD_*, 0). The bubble *B* has a refractive index *n_B_*, absorption coefficient *α_B_*, and circular shape with diameter *d_B_* (where *d_B_* < *x_PhD_*), and is positioned between the emitter and the photodetector in (*x_B_*, *y_B_*), since (*d_B_*/2) < *x_B_* < (*x_PhD_* − *d_B_*/2). The medium *M* where the bubble flows is considered homogeneous, has a refractive index *n_M_*, and an absorption coefficient *α_M_.*

When an emitted ray intercepts the bubble at (*x*_1_, *y*_1_), the attenuation caused by the medium *M* is first calculated through Beer-Lambert’s law [20]:

(1) $$I=I_{0}e^{-\alpha lc}$$

In the above equation, *I* is the final intensity, *I*_0_ the initial intensity, *α* the absorption coefficient, *l* the optical path length, and *c* the concentration of the medium.

Secondly, still at the point (*x*_1_, *y*_1_), the ray undergoes refraction which is calculated by Snell’s law [21]:

(2) $$\theta_{r}=\sin^{-1}\left(\frac{n_{i}\cdot \sin\theta_{i}}{n_{r}}\right)$$

where *θ_r_* is the refraction angle, *n_i_* is the refractive index of the incident medium, *θ_i_* is the incidence angle, and *n_r_* is the refractive index of the refraction medium.

The intensity of the ray decreases as part of it is reflected and another part refracted. The intensity of the refracted ray component is given by the Fresnel’s coefficients [21]:

(3) $$\begin{array}{l} T_{par}=4\frac{\sin\left(\theta_{i}\right)\sin\left(\theta_{r}\right)\cos\left(\theta_{i}\right)\cos\left(\theta_{r}\right)}{\sin^{2}\left(\theta_{i}+\theta_{r}\right)\cos^{2}\left(\theta_{i}-\theta_{r}\right)};\\ T_{per}=4\frac{\sin\left(\theta_{i}\right)\sin\left(\theta_{r}\right)\cos\left(\theta_{i}\right)\cos\left(\theta_{r}\right)}{\sin^{2}\left(\theta_{i}+\theta_{r}\right)} \end{array}$$

where *T_par_* and *T_per_* are the ray coefficients for the refracted ray when the incident ray is decomposed into parallel and perpendicular components, respectively. Considering that on average the difference between the parallel and perpendicular components is <0.5%, only the perpendicular coefficient is considered.

The refracted ray at the interface in the point (*x*_1_, *y*_1_) propagates inside the bubble until it intersects the interface bubble medium at position (*x*_2_, *y*_2_), where *x*_2_ > *x*_1_. Again, the attenuated intensity is calculated through Equation (1), the angle of refraction by Equation (2), and the refracted intensity by Equation (3).

**Figure 3:**
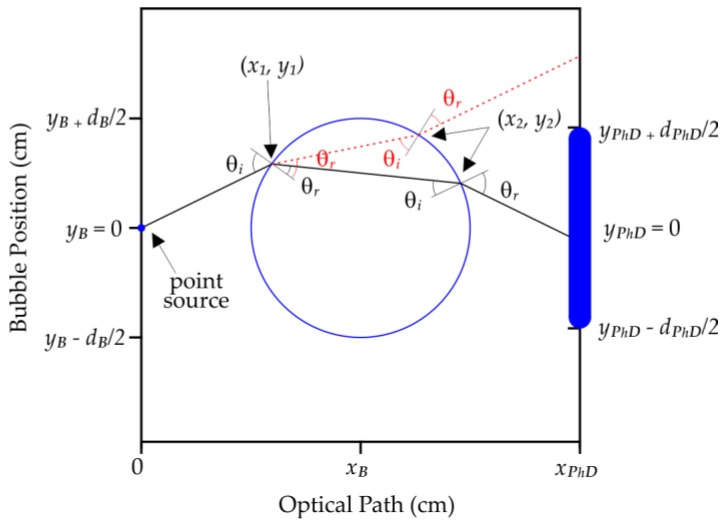
Schematic of the propagation of a single ray analyzed by the proposed mathematical model in two situations: the solid line arises on the photodetector and the dashed line does not. The design is shown without scale in order to exemplify the method.

The refracted ray at the interface at (*x*_2_, *y*_2_) propagates through the medium *M* until it intercepts the detector. Between the intersection (*x*_2_, *y*_2_) and the photodetector at (*x_PhD_*, *y_PhD_*), being (−*d_PhD_* ≤ *y_PhD_* ≤ *d_PhD_*), the attenuation is calculated by Equation (1). The total intensity detected is the result of the sum of the intensities of the rays that intercept the photodetector. Figure 3 shows schematically the path single ray as it interacts with a bubble.

**Figure 4:**
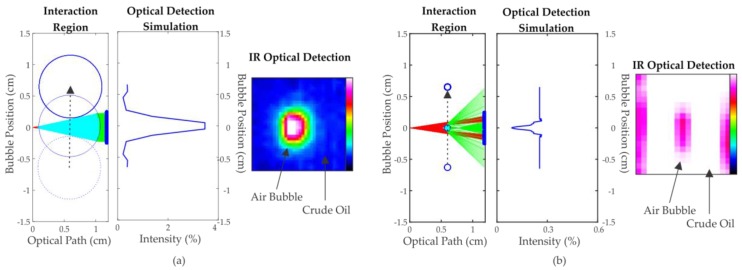
Simulation of the evolution of air bubbles flowing in crude oil (*n_M_* = 1.5; *α_M_* = 2.3 cm^−1^). (**a**) *d_B_* = 10 mm; *n_B_* = 1; *α_B_* = 0; (**b**) *d_B_* = 1 mm; *n_B_* = 1; *α_B_* = 0.

Results of the simulation for air bubbles with a diameter (*d_B_*) of 10 mm (*n_B_* = 1; *α_B_* = 0) in crude oil (*n_M_* = 1.5 [22]; *α_M_* = 2.3 cm^−1^ [23]) are observed in Figure 4a. Because of the low absorption of the air in comparison to the oil, an increase in the intensity of the signal detection is expected during the bubble flow. The maximum intensity occurs when the bubble center is aligned with the center of the optical path, as can be observed on the optical detection simulation and the IR optical detection.

Figure 4b shows the result of the evolution of one air bubble with a diameter of 1 mm. In this case, unlike the previous situation, instead of showing a higher detection intensity, especially when the bubble is in the middle of the optical path, the simulation shows a low intensity signal detection. This simulation result is validated by the IR optical detection, as shown in Figure 4b. In this case, the effect of refraction is greater than the absorption.

**Figure 5:**
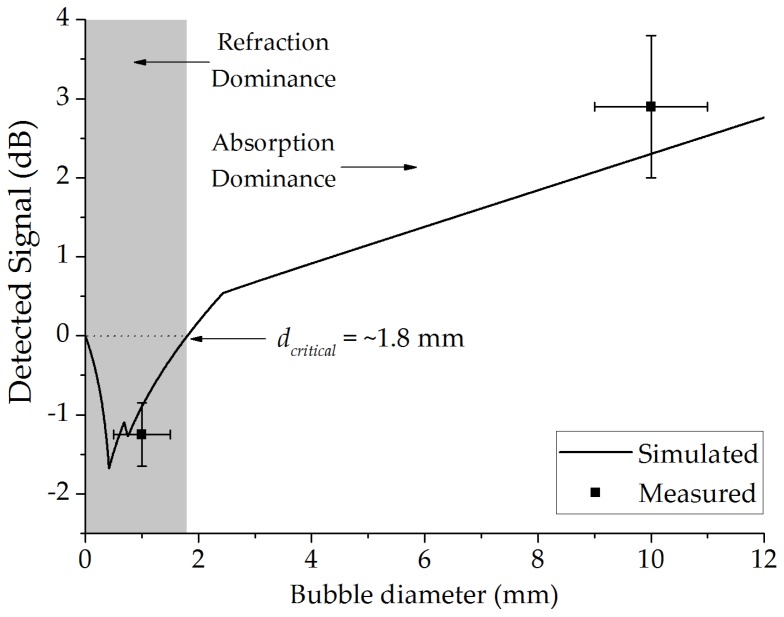
Detected signal as a function of the bubble diameter.

The plot in Figure 5 shows the refraction or the absorption effects as a function of the air bubble diameter (*d_B_*). A critical diameter of ~1.8 mm is obtained using the mathematical model, which shows the limit for the dominance of absorption or refraction. For comparison, the mean of the performed measurements is also plotted with air bubbles flowing in crude oil with average diameters of 1 and 10 mm. All data corresponds to the bubble at the center of the optical path. The reference of the detected signal is the optical path filled with crude oil.

It can be observed that the refraction is more significant than the absorption up to a diameter of 0.4 mm, causing the decreasing of the signal intensity with the increase of the bubble diameter. From 0.4 mm, the absorption begins to have significant role, increasing the signal intensity detected up to 1.8 mm. From 1.8 to 12 mm, we have the dominance of the absorption.

Error bars are calculated from the standard deviation between measurements. The difference between the simulated and measured signal can be related to the absorption coefficient and refractive index of crude oil used in the numeric model. Given that crude oil is a complex substance, these values can vary significantly depending on the sample.

### 3.3. Air Bubbles Flow through Static Crude Oil

**Figure 6:**
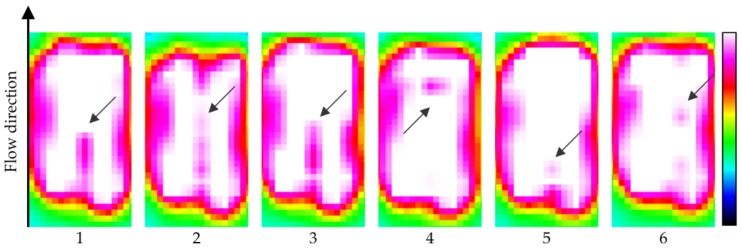
Frames of six different small bubbles flowing in static crude oil. The arrows show the location of the bubbles.

The experiment is performed by manually injecting air bubbles into static oil inside a cuvette. The diameter of the droplets is controlled by the volume and speed of the air injection. All IR images acquisitions are performed in region (B″) of Figure 2. Frames with IR images of distinct bubbles (average diameter of 1 mm) flowing in crude oil are observed in Figure 6. Here the bubbles are detected by pink (lower intensity) spots in the white (high intensity) area which denotes the optical window containing the crude. It was not possible to calculate the air bubbles velocity due to the camera's low acquisition speed (10 Hz). These results confirm the data generated by the simulation, where the phenomenon of refraction is predominant on the effect of absorption when the bubbles have a small diameter.

**Figure 7:**
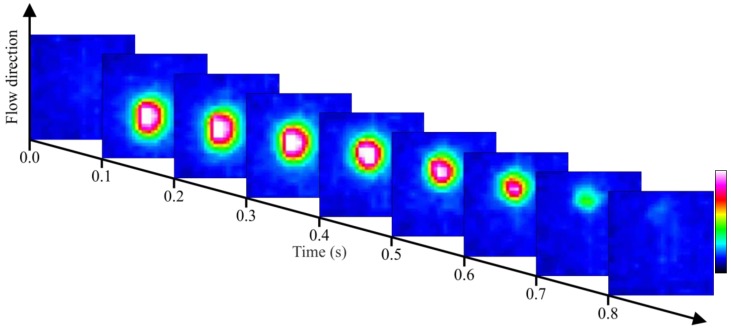
Flow of one air bubble through the static crude oil.

Another test is performed with the static crude oil and the air flowing, but this time with large air bubbles (*d_B_* = ~10 mm) (Figure 7). The optical detection system is in thermal equilibrium, operating in the region (B″) presented in Figure 2. In this test, the crude oil sample had its volatile components evaporated and have a higher viscosity and density, thereby decreasing the air flow velocity and facilitating the acquisition of images. Since air has a lower absorption than crude oil, combined with the reflection and refraction effects of large diameter bubbles, this results in a more intense signal that reaches the sensor. The measured air bubble velocity is of 30 mm/s and is calculated based on the acquisition rate of the sensor and the displacement of the air bubble.

### 3.4. Crude Oil and Air Flow

**Figure 8:**
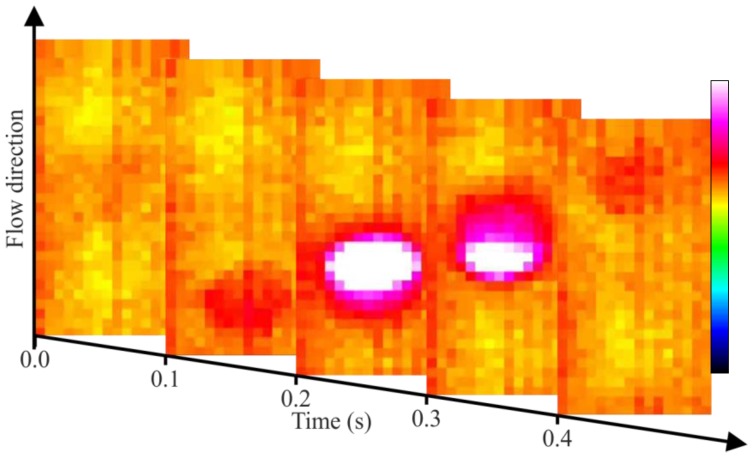
Two-phase flow involving air and crude oil.

For this test, the crude oil is mechanically pumped at approximately 58 mm/s through the entire loop. Near the interaction region, bubbles of air are manually injected. The data acquisition occurs when the optical system is operating in region (B″) of Figure 2. The air flow in flowing crude oil is observed frame by frame in Figure 8. The calculated velocity of the air bubbles is estimated to be 46 mm/s.

It is possible to distinguish the presence and direction of the bubble flow, but it is not possible to visualize the shape of the drop carrying out a visual characterization of the flow pattern (bubble, slug, churn, annular, etc.).

## 4. Conclusions

In this paper, the implementation of an optical detection system to visualize the flow of gas bubbles in crude oil by an infrared technique was presented. The initial results are promising in spite of the acquired images having low resolution and low acquisition rate. For the multiphase flows, it has the advantage of being nonintrusive, and thus does not influence the behavior of the flow. Given the passage of radiation through the crude oil sample, it starts to slowly heat up, but not enough to cause combustion of the oil.

Images are achieved using infrared radiation through a 12 mm length of crude oil. This infrared technique also presents the possibility for real-time monitoring of two-phase flows even without image processing.

The presented flow detection technique should result in the development of instrumentation for potential application in the field for characterizing flows involving petroleum and other substances such as water, CO_2_, H_2_S, natural gas and others. For the detection of other substances, the same principle presented here can be used, it is only needs the choice of appropriate wavelengths. The choice of wavelength operating regions must be made so that all the substances involved have different absorptions, as in the case of crude oil, water and air at the region between 8 and 12 μm. Furthermore, the present low-resolution optical system can be seen as a proof-of-concept for the development of high spatial and temporal resolution systems (similar to current high-speed cameras [24]) for multiphase flow investigation, either as laboratory tool or for field applications.

## Figures and Tables

**Table 1:** FTIR transmittances for different spectrum regions of the crude oil sample.

	Region 1	Region 2	Region 3	Region 4	Region 5
Wavelength	0.5 μm	3.4 μm	4–6 μm	7 μm	8–12 μm
Transmittance	<0.1	<0.4	>0.9	0.7	>0.9
